# Effect of Marriage on Burnout among Healthcare Workers during the COVID-19 Pandemic

**DOI:** 10.3390/ijerph192315811

**Published:** 2022-11-28

**Authors:** Yong-Hsin Chen, Shu-Zon Lou, Ching-wen Yang, Hsiu-Mei Tang, Chiu-Hsiang Lee, Gwo-Ping Jong

**Affiliations:** 1 Department of Public Health, Chung Shan Medical University, Taichung 40201, Taiwan; 2 Department of Occupational Safety and Health, Chung Shan Medical University Hospital, Taichung 40201, Taiwan; 3 Department of Occupational Therapy, Chung Shan Medical University, Taichung 40201, Taiwan; 4 Occupational Therapy Room, Chung Shan Medical University Hospital, Taichung 40201, Taiwan; 5 Department of Nursing, Chung Shan Medical University, Taichung 40201, Taiwan; 6 Department of Nursing, Chung Shan Medical University Hospital, Taichung 40201, Taiwan; 7 Department of Internal Medicine, Chung Shan Medical University Hospital and Chung Shan Medical University, Taichung 40201, Taiwan

**Keywords:** personal burnout, work-related burnout, COVID-19, marriage, parenthood, mediation effect, suppression effect

## Abstract

Since the onset of the COVID-19 pandemic, burnout symptoms have been prevalent among healthcare workers. Living with spouses can be complex and was associated with an increased burnout risk during the COVID-19 pandemic. This study investigated the relationship between living with spouses and burnout among healthcare workers during the COVID-19 pandemic. We distributed questionnaires to participants working in a hospital affiliated with a medical university in Taiwan. The questionnaires were the Copenhagen Burnout Inventory, which comprises personal burnout (PB), work-related burnout (WB), and client burnout subscales; the Nordic Musculoskeletal Questionnaire; and information on basic demographic variables, family factors, living habits, work-related factors, and physical health factors. Multiple linear regression and mediation analysis were used. We obtained 1615 (63.81%) valid questionnaires. After analysis revealed that marriage was an independent risk factor for PB; however, the effect of marriage on WB was nonsignificant after controlling for risk factors. Parenthood, less alcohol use, reported sleep duration less than six hours, less overtime, less shift work, and participation in leisure activities with family and friends were found to be mediators between marriage and a lower WB level. In addition, chronic diseases, frequent neck pain, and shoulder pain were suppression factors. In summary, marriage was associated with an increased risk of PB. Married individuals sustain a high WB level because of changes in family roles, living conditions, and work conditions. Overall, helping healthcare workers to maintain well-being in marriage or family living may be effective in decreasing burnout during the COVID-19 pandemic.

## 1. Introduction

Burnout was first described in 1974 by clinical psychologist Herbert Freudenberger [1]. The Maslach Burnout Inventory [2] and the Copenhagen Burnout Inventory (CBI) [3] have been demonstrated to be appropriate and effective burnout measurement tools in healthy populations [4]. Burnout was recognized in the *International Classification of Diseases, 11th Revision*, as an “occupational phenomenon” [5]. It was defined as a state of physical, emotional, and mental exhaustion that results from long-term involvement in work situations that are emotionally demanding [6]. Clinically, severe burnout can present as emotional exhaustion, physical fatigue, cognitive impairment, disturbed sleep, and functional impairment [7,8]. The stressors often contribute to clinical burnout [9].

Burnout is common among health-care providers [10,11,12]. Burnout causes high physician turnover and reduced clinical hours, which cause a total loss of approximately US$4.6 billion each year [13]. Factors associated with burnout include sleep duration on working days [14], musculoskeletal pain [15], increased risk of injury [16], disturbance of home and family life [17], and chronic diseases such as coronary heart disease [18], and type 2 diabetes [19]. Burnout symptoms have been prevalent among health-care workers since the onset of the COVID-19 pandemic in 2020 [20]. Over 50% of health-care professionals reported burnout during the COVID-19 pandemic, mainly caused by contact with patients, supply shortages, and work affecting household activities [21].

The marriage was simultaneously a source of support and stress in adult life [22]. Social support is a major risk factor for mortality [23]. Married individuals reported greater happiness and life satisfaction than did unmarried individuals; among them, loneliness and isolation appear to be a substantial reason [24]. Thus, the satisfaction and support obtained from marriage or the relationship with the spouse benefit married individuals [25] and can buffer stressful relationships at work [26,27]. However, the marriage relationship and quality of life could be impaired by psychological distress [28], the death of a child [29], and economic stress [30], which could weaken the buffer effect between marriage and work stress.

Prepandemic studies have demonstrated that single, divorced, or unmarried individuals easily meet the criteria for burnout compared with those who are married [31]. In addition, the parent’s role in the family’s important relationship considerably mitigates burnout development [32]. During the COVID-19 pandemic, individuals who were single, widowed, or divorced seemed to be associated with higher PB than those who were married [33]. However, the same association was not observed in WB. According to the definition of CBI [3], PB is the degree of physical and psychological fatigue and exhaustion experienced by a person who must not consider their occupational state. WB is the degree of physical and psychological fatigue and exhaustion that is perceived by the person as related to work that has been paid in some kind of way. By comparing both scales of PB and WB, the fatigue due to nonwork factors (such as health or family demands) could be identified. In addition, the effect of living with spouses on burnout is multifaceted. Therefore, this study determined whether there is a difference between being married and developing PB or WB and to further explore the reasons for marriage impacting burnout under several stressors, such as family, the workplace, and taking care of patients.

## 2. Methods

This cross-sectional observational study was conducted during the COVID-19 pandemic. We distributed questionnaires to 2531 employees from a hospital affiliated with a medical university in Taichung, Taiwan, from March to April 2021. Of the 1633 (64.52%) responses received, 1615 (63.81%) were deemed valid. This survey comprised the Nordic Musculoskeletal Questionnaire, the CBI, and information on basic variables for demographic. The professional fields of 1615 healthcare workers were classified in Supplementary Information Table S1 and reclassified as physicians, nurses, professional and technical personnel, and administration staff.

We assessed the participants’ education level (“high school or lower”, “Bachelor’s degree”, “Master’s degree”, or “PhD and above”), marital status (“Married” or “Other”), number of children (“zero”, “one”, “two”, “three”, or “more than three”), with a nonzero number of children reclassified as the new variable “parenthood.” Relationships with family and friends were evaluated by asking if the participants engaged in leisure activities with family or friends (LAFF) during vacation time. The response options for a suitable parametric test 5-point Likert scale method [34] were “always”, “often”, “sometimes”, “rarely”, and “never”, with corresponding scores of 100, 75, 50, 25, and 0 points, respectively. The presence of one or more chronic diseases was also noted as “yes”. Alcohol use in the past month was rated as “always”, “often”, “sometimes”, “rarely”, and “never” corresponding to 100, 75, 50, 25, and 0 points, respectively. Responses for sleep duration were <5, 5–6, 6–7, 7–8, or >8 h per day, which were reclassified as <6 h and SLD >6 h per day. Responses for exercise habit were “at least once a day”, “at least once a week”, “at least once a month”, “less than once a month”, or “never”, and exercising at least once a day or week was reclassified as “regular weekly exercise.” The response options for overtime were “rarely”, “less than 45 h per month”, “45–80 h per month”, and “more than 80 h per month.” Responses other than “rarely” were reclassified as “experiencing overtime work.” The responses to the question on the shift schedule were “day shift work”, “night shift work”, “irregular shift work”, and “regular shift work.” The professional fields were classified as nurses, administration staff, physicians, including attending physicians and residents, and professional and technical personnel.

The Chinese version of the CBI is reliable and valid for the assessment of burnout problems [35,36]. The scales are listed in Supplementary Information Table S2. The response options—“always”, “often”, “sometimes”, “rarely”, and ”never/almost never”—were scored as 100, 75, 50, 25, and 0 points, respectively, except for item C13, which was inversely scored (i.e., 0, 25, 50, 75, and 100 points, respectively); the calculated mean values indicated the PB and WB level for the participants.

MS pain was considered a confounder of burnout due to the close correlation between musculoskeletal pain and burnout [15,37]. Therefore, MS pain must be one of the adjusted variables in a multiple linear regression model on burnout. We adopted the Nordic Musculoskeletal Questionnaire, modified and translated by the Taiwan Institute of Occupational Safety and Health [38], to determine if the presence of pain was attributable to work-related factors in the preceding year. The response options on pain frequency are: every day, once a week, once a month, once every half year, or at least once every half year, corresponding to 100, 80, 60, 40, and 20 points, respectively. We used factor analysis [39] to determine new underlying variables to effectively explain the questionnaire. The results and calculation process are presented in Supplementary Information Table S3. The redefined underlying variables were neck and bilateral shoulder pain, both ankle pain, and bilateral knee pain, respectively.

The *t* test and one-way ANOVA were adopted for testing the difference on the dependent variable among two or more independent variables. The univariate and multiple linear regression models were adopted for the model. Whether a mediation effect existed among independent variables, the dependent variable, and the mediation factor was determined using the strategy proposed by Baron and Kenny [40], in which, (1) for the first-stage effect, the independent variable significantly affects the mediator, (2) the independent variable significantly affects the dependent variable in the absence of the mediator, (3) for the second-stage effect, the mediator exerts a significant unique effect on the dependent variable, and (4) the effect of the independent variable on the dependent variable weakens upon the addition of a mediator to the model.

A mediation model suitable for the combination of categorical and continuous variables was developed by Iacobucci (2012) [41]. The formulas are listed in Supplementary Information Table S4. The traditional mediation effect is generally recognized as a, b, c, and c’ in the same direction. A study indicated that when a×b has the opposite sign to that of c’, a×b is an estimate of the suppression effect [42], the statistical significance of which can be verified using the Sobel test.

## 3. Results

Table 1 and Table 2 primarily identified risk factors for PB and WB. These risk factors for PB and WB would be added as controlled variables in multiple linear regression models. According to the strategy proposed by Baron and Kenny [40], the mediation factor must satisfy the condition that an independent variable is significantly associated with the mediation factor. Therefore, Table 3 and Table 4 mainly determined if the association between marriage state (an independent variable) and a possible mediation factor (the risk factor of PB or WB) were significant in statistics. Table 5 presents the multiple linear regression models of the marriage effect on PB and WB in the absence and presence of confounders. These models could determine if marriage states independently affect PB and WB. The mediation model was established in Table 6, which could find the mediation factors of the marriage effect on burnout.

### 3.1. Finding Risk Factors for PB and WB

Significant differences were observed in PB among exercise habits (*p* < 0.0001), alcohol use habits (*p* < 0.0001), sleep duration per day (*p* < 0.0001), overtime work state (*p* < 0.0001), shift schedules (*p* < 0.0001), profession (*p* < 0.0001), and chronic diseases (*p* < 0.0001; Table 1). A lower PB level was associated with increased LAFF (B = −0.12, *p* < 0.0001) and age (B = −0.25, *p* < 0.0001). A higher PB level was associated with increased neck and bilateral shoulder pain (B = 8.25, *p* < 0.0001), bilateral ankle pain (B = 1.46, *p* = 0.006, and bilateral knee pain (B = 1.98, *p* = 0.001; Table 2). Significant differences were observed in WB between marital status (*p* < 0.0001), parenthood (*p* < 0.0001), exercise habit (*p* < 0.0001), alcohol use habit (*p* < 0.0001), sleep duration per day (*p* < 0.0001), education level (*p* = 0.034), overtime work state (*p* < 0.0001), shift schedules (*p* < 0.0001), profession (*p* < 0.0001), and the presence of chronic disease (*p* < 0.0001; Table 1). A lower WB level was associated with increased LAFF (B = −0.14, *p* < 0.0001) and age (B = −0.31, *p* < 0.0001). Higher PB and WB levels were associated with an increased score in the neck and bilateral shoulder pain (B = 8.25, 6.32; both *p* < 0.0001), bilateral ankle pain (B = 1.46, *p* = 0.006; B = 1.42, *p* = 0.003), and bilateral knee pain (B = 1.98, *p* = 0.001; B = 1.31, *p* = 0.013; Table 2).

### 3.2. Finding Possible Mediation Factors of Marriage Effect on Burnout

Table 3 indicates significant differences in marital status among sexes (*p* = 0.003), parenthood statuses (*p* < 0.0001), exercise habits (*p* = 0.049), alcohol use habits (*p* = 0.012), sleep duration per day (*p* = 0.004), education levels (*p* < 0.0001), overtime work states (*p* = 0.000), shift schedules (*p* < 0.0001), professions (*p* < 0.0001), and the presence of chronic diseases (*p* = 0.004).

Married individuals had higher LAFF values (60.40 ± 19.95 vs. 53.47 ± 20.21, *p* < 0.0001), were older (43.44 ± 8.73 vs. 33.35 ± 9.00, *p* < 0.0001), and more often had neck and bilateral shoulder pain (0.07 ± 0.96 vs. −0.07 ± 0.87, *p* = 0.003) than did those with “other” marital status (Table 4).

### 3.3. To Establish the Linear Regression Models of Marriage Effect on Burnout

Three models—M_0_, M_1_, and M_2_—were established (Table 5). M_0_ was a univariate linear regression for PB/WB (dependent variable) and married (independent variable). M_1_ and M_2_ were multiple linear regression models for PB/WB (dependent variable) and married (independent variable), with control for the variables affecting PB/WB in Table 1 and Table 2. In the M_2_ model for WB, two dummy variables—married and parenthood—could create multicollinearity in the multiple regression because 93.3% of the married individuals were also parents (Table 3). As a result, two dummy variables would not contribute to the M_2_ model together.

In the M_0_ model for PB and WB, marriage was not associated with a lower PB level (B = −1.06, *p* = 0.240); however, marriage was significantly associated with a lower WB level (B = −4.30, *p* < 0.0001). In M_1_ and M_2_ for PB and WB, marriage was significantly associated with a lower PB level (B = 2.12, *p* = 0.018) and not associated with a lower WB level (B = −1.05, *p* = 0.201). Marriage was an independent risk factor for PB, and the effect of marriage on WB can be explained by other risk factors for WB (Table 5).

### 3.4. To Establish the Mediation Models of Marriage and Their Effect on Burnout

The effect of marriage on WB could be explained by other risk factor effects on WB (Table 5). To further explore, we modeled marriage, the mediation factor, and WB. The mediation models used the risk factors for WB that were significantly associated with marital status as possible candidates for mediation factors. Parenthood, exercise habits, alcohol use, sleep duration < 6 h, experience with overtime, irregular work shifts, LAFF, and neck and bilateral shoulder pain were added to the candidate list. Table 6 summarizes the results of the mediation models for the candidate variables.

Parenthood (Z = −2.89, *p* < 0.01), alcohol use (Z = −2.12, *p* < 0.05), sleep duration < 6 h (Z = −2.67, *p* < 0.01), experience with overtime (Z = −3.34, *p* < 0.01), shift work (Z = −4.84, *p* < 0.0001), and LAFF (Z = −4.74, *p* < 0.0001) were factors that mediated the relationship between marriage and lower WB levels. The changes due to living with spouses affect family roles, living habits, work hours and schedules, and social relationships.

### 3.5. The Marriage Effect on Burnout

We found that marriage was an independent risk factor for PB; however, the marriage effect on WB could be explained by other risk factors. Notably, parenthood, ever alcohol use, sleep duration less than 6 h per day, experience of overtime, shift work, and LAFF were all found to be mediators of the marriage effect on WB.

## 4. Discussion

Through multiple linear regression, we explored the relationship between marriage and burnout during the COVID-19 pandemic and discovered that marriage was associated with increased PB. Married individuals reported low WB, which may be attributed to the parent’s role, reduced alcohol use, frequent sleep duration of more than 6 h, rare working overtime, rare having shift work, and frequent participation in leisure activities with family and friends when on vacation.

Individuals engaging in regular exercise had low levels of PB and WB (all *p* < 0.001; Table 1). The physiological changes due to physical activity may reduce physiological sensitivity to chronic stress, which could lead to faster physical recovery from stressful situations and a reduced burnout risk [43,44,45,46].

The frequent alcohol consumers had higher levels of PB and WB than those who rarely consumed alcohol in a month (Table 1 and Table 2). Whether alcohol use reduces stress is debatable [47,48]. In addition, burnout was strongly associated with alcohol abuse or dependence [49,50].

Individuals with a sleep duration of <6 h and those who work overtime or in shifts had a higher risk of PB/WB than others (Table 1 and Table 2). Sleep duration of <6 h was correlated with self-reported stress [51] and higher risks of burnout [14]. Overtime and shift work are related to short or disturbed sleep, which could also cause high levels of burnout [52,53,54,55,56,57,58].

Studies have identified that work-related stressors, high workload, and work–home conflict are associated with burnout among physicians [59,60,61,62]. Notably, burnout remains markedly more prevalent among practicing physicians than among individuals in other fields after adjustment for work hours and other factors [63,64]. According to our data, physicians have higher PB and WB levels than nurses, professionals, technical personnel, and administration staff (Table 1 and Table 2).

Burnout was associated with an increased risk of musculoskeletal pain and can predict the onset of regional neck/shoulder and lower back pain [37]. We discovered that frequent neck and bilateral shoulder pain was associated with an increased risk of PB and WB (both *p* < 0.001; Table 1 and Table 2).

Family members and the support of friends and colleagues play a vital role in alleviating burnout [17,65]. In addition, LAFF relieves stress, helps individuals cope with emotions, and moderately maintains physical and mental health [66,67]. Positive engagement in LAFF was associated with a low level of PB and WB among our participants (Table 4).

Marriage was significantly correlated with a high level of PB in the presence of adjusted variables (Table 5; M1; B = 2.12, *p* < 0.05). PB is the degree of physical and psychological fatigue and exhaustion [3], which excludes factors from the workplace and the client. In our cohort, 93.31% of married individuals had ≥1 children (Table 3). This implies that the effect of marriage on PB can be explained by the effect of parenthood. Parenthood is complex and stressful, which exposes parents to chronic stress known as “parental burnout” and it has been reported that there are significant and positive correlations between parental burnout and professional burnout [68]. Therefore, raising children in marriage relationships plays a vital role in worsening PB. Our study was conducted during the COVID-19 pandemic. Studies have demonstrated that in addition to lower PB levels among single people during the COVID-19 pandemic than among married people, disturbances in home and family lives were correlated with burnout [17,33]. A study from Taiwan during the COVID-19 pandemic revealed the following fears among medical staff: (1) passing on COVID-19 to relatives and friends; (2) being separated from family; and (3) the inconvenience of taking care of children or family members [69]. These concerns may also explain the high levels of PB among married individuals in our cohort.

Individuals with married status would consume alcohol in moderation [70]. The married co-twins consumed fewer alcoholic beverages than did their single or divorced co-twins [71]. On the basis of the association between marriage and decreased alcohol use (Table 3), we used the mediation model in Table 5 to determine that decreased alcohol use is a reason that married individuals have a low WB level (Z = −2.12, *p* < 0.05).

Studies have shown that unmarried individuals reported sleep duration that was 1.53 times shorter than that of married individuals, an average of 4.8 min less [72,73]. Our data indicate that unmarried individuals reported sleep durations that were 1.35 times shorter than those of married individuals (Table 6). In addition, the mediation model demonstrated that a low proportion of sleep duration < 6 h is a reason why married individuals have lower WB levels than those with “other” marital status (Z = −2.76, *p* < 0.01).

Although marriage is associated with longer lifespans [74], our data indicate that married individuals exhibited a higher proportion (52.66% vs. 45.34%) of chronic disease than did those with the “other” marital status. The presence of chronic disease suppressed the relationship between marriage and decreased WB (Z = 2.42, *p* < 0.01; Table 6).

Participation in family leisure led to improved interactions and cohesion within families and improved parents’ and children’s well-being, which is integral to well-functioning marriages [75,76,77]. Our mediation model (Table 6) indicated that the increased frequency of leisure activities with family and friends was also a reason for the marriage effect on decreased WB (Z = −4.74, *p* < 0.001).

Married workers have a higher risk of musculoskeletal pain than unmarried, divorced, widowed, and separated workers [78]. Married individuals reported a higher frequency of neck and bilateral shoulder pain than those with the “other” marital status (Table 4). However, the mediation model (Table 6) revealed that sustained neck and bilateral shoulder pain was a suppression factor, and married individuals have low levels of WB. Low mood or stress at baseline is more likely to result in neck and shoulder pain, indicating that married individuals may experience more stress than those with the “other” marital status [79].

We discovered that marriage affected PB and WB differently during the pandemic. Specifically, marriage and family living were attributed to severe PB. This may be because, during the pandemic, married healthcare workers may additionally worry about their family or children [69]. However, according to mediation models, marriage and living with a spouse alleviated WB during the pandemic. According to the theory of work–family enrichment [80], participation in one role may enrich the quality of life in the other. The previous research had determined that work and family identities and support from the family were one of the individual, work, and family antecedents of enrichment [81]. In addition, participation in family leisure could improve interactions, family cohesion, functioning, and satisfaction [75,82], which are also integral to well-functioning marriages [77]. The mediation models showed that raising children, less alcohol use, longer sleep duration, less overtime, less shift work, and being positively engaged in LAFF are the main reasons that married individuals sustain low WB. We believe these mediation factors hint that individuals were positively trying to play a successful family role, which could further enhance the quality of life of another role played, such as staff. This could explain why married individuals sustain lower WB than those who are not married.

Therefore, strong relationships with spouses and family members are necessary to alleviate burnout, especially the burnout that results from work-related factors. We suggest that well-being after marriage and positively taking a competent role (wife or husband) in a marriage are reasons that the effects of marriage on burnout differ. This is a key finding that has been rarely discussed in other studies.

We did not collect data on the age of the participants’ children, which can affect individuals’ stress levels. To overcome this limitation, we controlled for the participants’ age. We also did not collect data on parental and marital burnout, stress, or satisfaction; these factors may affect the relationship between marital status and burnout. Work–family conflict results from a psychological phenomenon of imbalance between work and home life, which is an important model for exploring the relationship between family and work. Since the present study did not collect related data using the Measure of Work–Family Conflict Questionnaire, we are unable to further explore the marriage effect on burnout using the work–family conflict model. In addition, multigenerational families and taking care of elderly parents are common in Taiwanese society, but this study did not explore these factors.

The means of PB and WB were 36.09 ± 18.05 and 34.21 ± 16.25 in the present study, respectively. A study regarding burnout among employees of a Malaysian hospital in 2018 demonstrated the means for PB and WB were 36.27 ± 19.21 and 32.87 ± 18.35 [83]. In addition, the measured means of PB and WB in India [84] and Lithuania [85] during the COVID-19 pandemic were 49.72 ± 18.68, 39.69 ± 20.43, 45.27 ± 17.77, and 46.41 ± 17.16. Notably, an anonymous self-administered questionnaire broadcasted via WhatsApp and Twitter for healthcare workers during the COVID-19 pandemic demonstrated that the means of PB and WB even reached 67.23 ± 21.66 and 61.38 ± 21.60 [86]. Obviously, different countries and regions may experience varying levels of overall burnout among due to differences in medical culture and environment. Therefore, the same study should be conducted in different countries for further comparison and to explore the reasons for differences in burnout. Although people who complete the the burnout questionnaire are not necessarily at risk for clinical burnout [8], people with short-term stress have higher levels of burnout [87], shorter recovery duration, and a more favorable prognosis compared to those with clinical burnout [88]. Therefore, CBI is still an effective tool for early warning of clinical burnout and stress.

## 5. Conclusions

In conclusion, we found that marriage was an independent risk factor for PB during the COVID-19 pandemic. In addition, the protective effect of marriage on WB is mainly attributed to the parental role that emerges after marriage, changes in living habits and health, decreasing amounts of overtime and shift work, and positive engagement in LAFF. Overall, maintaining marital well-being and healthy relationships with their family is the most effective strategy for decreasing burnout during pandemics and other high-workload situations. Specifically, an employee health promotion program should assist employees in breaking bad habits by elastically adjusting their work schedule, encouraging family leisure activities, and positively improving their relationships with their spouses and children.

## Figures and Tables

**Table 1:** Mean of PB and WB on Survey Variables.

		PB		WB	
Survey Variables	N	Mean ± SD	*p*	Mean ± SD	*p*
*-**Categorical variable**-*					
All individuals	1615	36.09 ± 18.05	-	34.21 ± 16.25	-
***Marriage state***					
Married	779	35.54 ± 17.68	0.240 ^†^	31.99 ± 15.38	<0.0001 ^†^
Other	836	36.60 ± 18.38		36.29 ± 16.76	
***Sex***					
Women	1314	36.43 ± 17.74	0.111 ^†^	34.55 ± 16.21	0.083 ^†^
Men	301	34.59 ± 19.31		32.75 ± 16.37	
***Parenthood***					
Yes	703	35.49 ± 18.51	0.243 ^†^	31.47 ± 15.83	<0.0001 ^†^
No	912	36.55 ± 17.68		36.33 ± 16.25	
***Exercise habit***					
Weekly exercise habit	933	33.43 ± 17.18	<0.0001 ^†^	31.85 ± 15.95	<0.0001 ^†^
Not weekly exercise habit	682	39.73 ± 18.59		37.45 ± 16.10	
***Alcohol use habit***					
Ever AU in a month	609	38.47 ± 17.68	<0.0001 ^†^	36.40 ± 15.67	<0.0001 ^†^
Never AU in a month	1006	34.65 ± 18.13		32.89 ± 16.45	
***Sleep duration per day***					
<6 h	626	41.07 ± 18.84	<0.0001 ^†^	38.05 ± 17.01	<0.0001 ^†^
>6 h	989	32.94 ± 16.79		31.78 ± 15.26	
***Education degree***					
Master’s degree or above	297	36.15 ± 34.08	0.946 ^†^	32.41 ± 15.79	0.034 ^†^
University or below university degree	1318	36.07 ± 18.03		34.62 ± 16.33	
***Overtime work state***					
Experience overtime	561	42.40 ± 18.09	<0.0001 ^†^	40.01 ± 15.71	<0.0001 ^†^
Seldom overtime	1054	32.73 ± 17.11		31.13 ± 15.68	
***Shift schedules***					
Irregular shift work	192	42.71 ± 18.58 ^a^	<0.0001 ^§^	40.94 ± 16.50 ^a^	<0.0001 ^§^
Regular shift work	196	39.44 ± 19.21 ^b^		38.30 ± 16.73 ^a^	
Night shift work	166	36.32 ± 18.96 ^b^		35.31 ± 16.45 ^b^	
Day shift work	1061	34.24 ± 17.22 ^c^		32.07 ± 15.59 ^c^	
***Profession***					
Physicians	138	41.91 ± 20.15 ^a^	<0.0001 ^§^	39.67 ± 17.34 ^a^	<0.0001 ^§^
Nurses	613	40.37 ± 18.11 ^a^		38.23 ± 16.26 ^a^	
professional and technical personnel	283	33.80 ± 17.01 ^b^		31.95 ± 15.64 ^b^	
Administration staffs	581	31.30 ± 16.47 ^b^		29.78 ± 14.82 ^b^	
***The presence of chronic diseases***					
Yes	638	38.92 ± 18.14	<0.0001 ^†^	36.31 ± 16.57	<0.0001 ^†^
No	977	34.24 ± 17.75		32.84 ± 15.89	

SD, standard deviation; N, participants; ^†^, *t*-test; ^§^, one-way ANOVA; ^a,b,c^, Means with the same letter are not significantly different (by Duncan’s multiple-range test). *p*, *p*-value.

**Table 2:** Univariate Linear Regression of Survey Variables against PB/WB.

	Individuals = 1615			
	Personal Burnout		Work-Related Burnout	
Survey Variable	B	*p*	B	*p*
LAFF	−0.12	<0.0001	−0.14	<0.0001
Age	−0.25	<0.0001	−0.31	<0.0001
Neck and both shoulders pain	8.25	<0.0001	6.32	<0.0001
Both ankles pain	1.46	0.006	1.42	0.003
Both knees pain	1.98	0.001	1.31	0.013

B, unstandardized linear regression coefficient; *p*, *p* value.

**Table 3:** Contingency Table Analysis for Categorical Variables and Marital Status.

Survey Variables	Married (%)	Other (%)	*p*
All individuals	779 (48.24)	836 (51.76)	-
***Sex***			
Women	610 (46.42)	704 (53.58)	0.003 ^†^
Men	169 (56.15)	132 (43.85)	
***Parenthood***			
Yes	656 (93.31)	47 (6.69)	<0.0001 ^†^
No	123 (13.49)	789 (86.51)	
***Exercise habit***			
Weekly exercise habit	470 (50.38)	463 (49.62)	0.049 ^†^
No weekly exercise habit	309 (45.31)	373 (54.69)	
***Alcohol use habit***			
Ever AU in a month	269 (44.17)	340 (55.83)	0.012 ^†^
Never AU in a month	510 (50.70)	496 (49.30)	
***Sleep duration per day***			
<6 h	273 (43.61)	353 (56.39)	0.004 ^†^
> 6 h	506 (51.16)	483 (48.84)	
***Education degree***			
Master’s degree or above	187 (62.96)	110 (37.04)	<0.0001 ^†^
University or below university degree	592 (44.92)	726 (55.08)	
***Overtime work state***			
Experience overtime	235 (41.89)	326 (58.11)	0.000 ^†^
Seldom overtime	544 (51.61)	510 (48.39)	
***Shift schedules***			
Irregular shift work	71 (36.98)	121 (63.02)	<0.0001 ^†^
Regular shift work	57 (29.08)	139 (70.92)	
Night shift work	38 (22.89)	128 (77.11)	
Day shift work	613 (57.78)	448 (42.22)	
***Profession***			
Physicians	62 (44.93)	76 (55.07)	<0.0001
Nurses	247 (40.29)	366 (59.71)	
Professional and technical personnel	173 (61.13)	110 (38.87)	
Administration staffs	297 (51.12)	284 (48.88)	
***The presence of chronic diseases***			
Yes	336 (52.66)	302 (47.34)	0.004
No	443 (45.34)	534 (54.66)	

*p*, *p*-value; ^†^, Fisher exact test.

**Table 4:** Leisure Activities, Age, and Pain Characteristics Stratified by Marital Status.

	Mean ± SD		
Survey variables	Married	Others	*p*
LAFF	60.40 ± 19.95	53.47 ± 20.21	<0.0001
Age	43.44 ± 8.73	33.35 ± 9.00	<0.0001
Neck and both shoulders pain	0.07 ± 0.96	−0.07 ± 0.87	0.003
Both ankles pain	−0.02 ± 0.79	0.02 ± 0.91	0.330
Both knees pain	−0.00 ± −0.75	0.00 ± 0.79	0.934

*p*, *p*-value; SD, standard deviation.

**Table 5:** Effect of Marriage on Burnout in the Presence or Absence of Confounders.

	Unstandardized Linear Regression Coefficient (B)							
	PB				WB			
Main Effect	M_0_ (*p*)	SE	M_1_ (*p*)	SE	M_0_ (*p*)	SE	M_2_ (*p*)	SE
Marriage	−1.06 (0.240)	0.90	2.12 (0.018)	0.90	−4.30 (<0.0001)	0.80	−1.05 (0.201)	0.82
adj. R^2^	0.00		0.31		0.02		0.28	

*p*, *p*-value; SE, standard error.; M_0_, Model in the absence of any confounder; M_1_, Model controlled for weekly regular exercise habits, previous alcohol use, sleep duration < 6 h, overtime, shift schedule, profession, the presence of chronic disease, LAFF, age, neck and bilateral shoulder pain, bilateral ankle pain, and bilateral knee pain. M_2_, Model controlled for education level of master’s degree or above but not for variables in M_1_.

**Table 6:** Mediation Models on the Relationship between Marriage and WB.

	The Mediation Effect between Marriage and WB					
Mediation Factor	c’	a	s_a_	b	s_b_	Z
Parenthood	−1.29	4.50 ***	0.18	−3.84 **	1.32	−2.89 **
Weekly exercise habit	−4.03 ***	−0.20 *	0.10	−5.40 ***	0.80	1.90
Ever alcohol use	−4.10 ***	−0.26 *	0.10	3.24 ***	0.83	−2.12 *
Sleep duration < 6 h	−3.87 ***	−0.30 **	0.10	5.98 ***	0.81	−2.76 **
Experience overtime	−3.55 ***	−0.39 **	0.11	8.53 ***	0.82	−3.34 **
Shift ^1^ work	−3.36 ***	−0.83 ***	0.12	6.43 ***	0.94	−4.84 ***
Suffering chronic disease	−4.57 ***	0.29 **	0.10	3.81 ***	0.82	2.42 **
LAFF	−3.40 ***	6.93 ***	1.00	−0.13 ***	0.02	−4.74 ***
Neck and both shoulders pain	−5.20 ***	0.14 **	0.05	6.53 ***	0.41	2.76 **

* *p* < 0.05; ** *p* < 0.01; *** *p* < 0.001; c’, direct effect; a, the linear or logistic regression coefficient of marriage against the mediation factor (first-stage effect); s_a_, the standard error of a; b, the linear regression coefficient of mediation factor against WB in the presence of the marriage factor (second-stage effect); s_b_, the standard error of b; ^1^, shift work includes irregular and regular shift work.

## Supplementary Materials

The following supporting information can be downloaded at: https://www.mdpi.com/article/10.3390/ijerph192315811/s1, Table S1: The professional field of participants; Table S2: The 13 items for the PB and WB scales; Table S3: Musculoskeletal Pain Sites and Factor Analysis of the Nordic Musculoskeletal Questionnaire; Table S4: The formulates for testing on mediation factor.
