# Supplementary material for: Effect of Marriage on Burnout among Healthcare Workers during the COVID-19 Pandemic
Source: Int J Environ Res Public Health. 2022 Nov 28;19(23):15811. doi: 10.3390/ijerph192315811 (PMC9737389; doi:10.3390/ijerph192315811)
Supplement: Supplementary file 1 [file ijerph-19-15811-s001.zip › ijerph-1924747-supplementary.pdf]

Supplementary Information

Table S1. The professional field of participants.

Professional field	individuals	%
Attending physician[1]	71	4.40
Resident physician[1]	67	4.15
Nurses	613	37.96
Respiratory therapist[2]	15	0.93
Physical therapist[2]	35	2.17
Social worker[2]	11	0.68
Nurse Practitioner[2]	53	3.28
Nutritionist[2]	15	0.93
Occupational Therapist[2]	31	1.92
Medical Radiation Technologist[2]	45	2.79
Medical technologist[2]	75	4.64
Psychologist[2]	3	0.19
Administration Staffs	581	35.98

[1], individuals were reclassified as Physicians; [2], individuals were reclassified as professional and technical personnel.

Table S2. The 13 items for the PB and WB scales.

	**The first six items, which concern PB**, are as follows:
1	"How often do you feel tired?"
2	"How often are you physically exhausted?"
3	"How often are you emotionally exhausted?"
4	"How often do you think 'I can't take it anymore?"
5	"How often do you feel worn out?"
6	"How often do you feel weak and susceptible to illness?"
	**Items 7–13, which concern WB**, are as follows:
7	"Is your work emotionally exhausting?"
8	"Do you feel burnt out because of your work?"
9	"Does your work frustrate you?"
10	"Do you feel worn out at the end of the working day?"
11	"Are you exhausted in the morning at the thought of another day at work?"
12	"Do you feel that every working hour is tiring for you?"
13	"Do you have enough energy for family and friends during leisure time?"

Table S3. Musculoskeletal Pain Sites and Factor Analysis of the Nordic Musculoskeletal Questionnaire.

MS pain site	MS pain subjects	Prevalence %	frequency score	Factor loading		
			mean ±SD	Factor 1	Factor 2	Factor 3
**Neck**	585	36.22	26.76±37.64	***0.33***	-0.02	-0.03
**Left shoulder**	325	20.12	15.07±31.62	***0.33***	-0.01	-0.01
**Right shoulder**	371	22.97	17.64±33.89	***0.33***	0.02	-0.07
Upper back	273	16.90	12.90±29.77	0.17	0.00	-0.01
Waist or lower back	451	27.93	20.20±34.72	0.08	-0.04	0.03
Left elbow	70	4.33	3.29±16.26	-0.05	-0.04	-0.05
Right elbow	113	7.00	5.33±20.43	-0.04	-0.04	-0.02

Left wrist	77	4.77	3.72±17.38	-0.05	0.00	0.01
Right wrist	162	10.03	7.51±23.66	-0.03	-0.03	-0.02
Left hip/thigh/buttock	67	4.15	3.12±15.64	-0.05	-0.07	-0.01
Right hip/thigh/buttock	68	4.21	3.17±15.83	-0.02	-0.04	-0.06
**Left knee**	80	4.95	3.78±16.98	-0.05	-0.07	***0.51***
**Right knee**	88	5.45	4.17±18.05	-0.02	-0.04	***0.45***
**Left ankle**	29	1.80	1.26±10.10	-0.02	***0.49***	-0.05
**Right ankle**	25	1.55	1.10±9.58	-0.02	***0.54***	-0.05
		eigenvalues		4.93	1.55	0.68
		explained variation %		57.59	18.12	0.08

Table S4. The formulates for testing on mediation factor.

If mediation factor and dependent variable were all continuous variables, the original formula of the Sobel test was applicable.

$$Z = \frac{a \times b}{\sqrt{b^2 {s_a}^2 + a^2 {s_b}^2}}$$

If mediation factor and dependent variable were categorical variables or a combination of categorical and continuous variables, the original formula of the Sobel test was rederived into a new formula.

$$(Z_m) = \frac{\frac{a}{s_a} \times \frac{b}{s_b}}{\sqrt{{Z_a}^2 + {Z_b}^2 + 1}}$$

a is a logistic or linear regression coefficient of independent variable against mediation factor; b is the logistic or linear regression coefficient of mediation factor against dependent variable in the presence of independent variable; The standard errors of a and b are represented by s_a and s_b, respectively. Z or Z_m exceeding ∣1.96∣, ∣2.57∣, and ∣3.90∣ (for a two-tailed test) were deemed significant at α = 0.05, 0.01, and 0.0001, respectively.
